# Extraction of nano-silicon with activated carbons simultaneously from rice husk and their synergistic catalytic effect in counter electrodes of dye-sensitized solar cells

**DOI:** 10.1038/srep39314

**Published:** 2016-12-21

**Authors:** Waqar Ahmad, Majid Raissan Al bahrani, Zhichun Yang, Jahangeer Khan, Wenkui Jing, Fan Jiang, Liang Chu, Nishuang Liu, Luying Li, Yihua Gao

**Affiliations:** 1 Center for Nanoscale Characterization & Devices (CNCD), Wuhan National Laboratory for Optoelectronics (WNLO) & School of Physics, Huazhong University of Science and Technology (HUST), Luoyu Road 1037, Wuhan 430074, P. R. China; 2 Center of Advanced Functional Ceramics (CAFC), Nanjing University of Posts and Telecommunications (NUPT), Nanjing 210046, P. R. China; 3 Hubei Collaborative Innovation Center for Advanced Organic Chemical Materials, 368 Youyi Avenue, Wuhan 430062, P. R. China

## Abstract

The extraction of renewable energy resources particularly from earth abundant materials has always been a matter of significance in industrial products. Herein, we report a novel simultaneous extraction of nano-silicon with activated carbons (nano-Si@ACs) from rice husk (RH) by chemical activation method. As-extracted nano-Si@ACs is then used as an energy harvesting materials in counter electrodes (CEs) of dye-sensitized solar cells (DSSCs). The morphology, structure and texture studies confirm the high surface area, abundant active sites and porous structure of nano-Si@ACs. Electrochemical impedance spectroscopy and cyclic voltammetry analyses reveal that the nano-Si@ACs is highly beneficial for fast I_3_^−^ reduction and superior electrolyte diffusion capability. The nano-Si@ACs CE based DSSC exhibits enhanced power conversion efficiency of (8.01%) in contrast to pristine Pt CE (7.20%). These favorable results highlight the potential application of RH in low-cost, high-efficiency and Pt-free DSSCs.

Rice (*Oryza sativa*) is the 2^nd^ leading staple food of the world. The threshing of rice lefts 20% husk as by-products of its total weight, so-called rice husk (RH)^1^,^2^. On average, RH contains 20% lignin, 55% holocellulose, 20% silica and 5% extractives and ashes^3^. As feedstock for animals, RH is unsuitable due to presence of enormous silica amount. Non-biodegradability of lignin content also makes its use incompatible as fertilizer^4^. Therefore, it is necessary to utilize the carbon contents or silica present in RH for commercial purposes.

Several attempts have been made to convert RH into industrial carbons so far^5^,^6^. Guo *et al*. reported that electro-chemical performances of activated carbons (ACs) obtained from RH can be developed in the absence of silica^7^. Since then, numerous routes have been designed to enhance the performances of ACs without silica content in DSSCs, super capacitors and energy storage devices^8^,^9^,^10^,^11^. Besides the ACs, silica content in RH may also play a key role in the preparation of various silicon-based functional materials^12^. Silicon (key component of silica) has interesting applications in electronics devices, energy harvesting and storage technology when its size is cut down to nanoscale^13^, even though it is highly reactive in electrochemical conditions and its surface features are strongly affected by environmental factors^14^. To improve reactivity and surface features for its use in commercial purposes, different passivation procedures have been adopted so far^15^,^16^,^17^,^18^,^19^,^20^. Beside their good efforts, there are so many drawbacks in the above extraction methods such as: (i) during the isolated extraction of ACs, Si is washed out and vice versa^8^,^20^; (ii) the individual extractions of ACs or Si from RH need further customization as an active materials for commercial purposes^16^,^17^; and (iii) more importantly the extraction of silica rather than Si strongly influences the electrochemical performance of photovoltaic and energy storage devices^21^.

The simultaneous extraction of Si with ACs from RH in a single step procedure is still key challenge. Although, simultaneous extraction of ACs and silica from RH have been reported^21^,^22^ but silica is non-significant as its applications concern in energy storage devices. Herein, we report the simultaneous extraction of ACs and Si (instead of silica) from raw RH in a single step via carbonization and magnesiothermic reduction process (as shown in Fig. 1) for DSSCs. Our extraction approach is very simple and beneficial in comparison to previous reported methods^16^,^20^,^21^,^22^ due to certain reasons: (i) the obtained nano-Si along with ACs have unique and remarkable structure, which is very promising for DSSCs performance; (ii) the entire procedure is time saving, energy efficient and easily extendable; and (iii) the whole process does not use any expensive fossil fuel (as carbon source) or bulk Si. Magnesium (Mg) is used here as reducing agent to decrease the reaction temperature for extraction of Si from ores of silica^23^. Furthermore, the metal Mg can regenerated as a byproduct MgCl_2_ by electrolysis^2^. The entire process consumes only HCl and converts it to Cl_2_ after the electrolysis. Furthermore, the recycling of RH into both ACs and Si is the first assessment for DSSCs to the best of our knowledge. We also collected nano-Si and ACs separately from RH as reported in literature for comparative analysis^2^,^8^. We doped some acetylene black into both extracted ACs and nano-Si@ACs to improve their low conductivity and amorphous structure. Particularly, under standard illumination condition (1.5 G, 100 mW cm^−2^) the as-synthesized nano-Si@ACs CE of DSSC showed 8.01% power conversion efficiency (PCE). We believe that enhanced performance is due to unique inter-linked structure of nano-Si with ACs, which may play a key role in future designing of photovoltaic devices.

## Results

### Material characteristics

Thermal gravimetric analysis (TGA) has great importance in knowledge contribution and was used to characterize the thermal behavior of biomass. The samples were heated in the temperature range of 30 to 800 °C @ 10 °C min^−1^ under nitrogen atmosphere. The TGA of RH used in our experiment verifies the presence of 22.9 wt% silica, as illustrated in Supplementary Fig. S1(a). In the TGA data, the amount of typical weight loss are recorded in three stages: (1) gentle weight loss in the temperature range of ~100–180 °C, which corresponds to the presence of bound water and moisture inside the sample; (2) weight loss in the temperature range of ~180–370 °C, which corresponds to the decomposition of cellulose and lignin of RH; and (3) weight loss occurs at above ~360 °C is due to the process of carbonization, which leads to the conversion of cellulose and lignin into gaseous materials and tars^24^. Furthermore, Williams *et al*. reported that lignin weight loss starts gradually in the temperature range of ~200–720 °C^25^. However, the TGA analysis of nano-Si@ACs composite exhibits the endothermic rapid weight loss up to 350 °C (see Supplementary Fig. S1(b)). The observed loss is attributed to the decomposition of carboxyl and lactonic groups present at the surface of ACs^26^. The drastic decrease in the mass of nano-Si@ACs within the temperature range from 350 to 550 °C, corresponds to the oxidation of carbons present in the specimen^27^. It is clear that the as-prepared nano-composite mostly contains carbon materials.

The surface morphology of final products obtained from RH was analyzed by FE-SEM. Figure 2(a) is the surface SEM micrograph of ACs extracted from RH separately. It can be seen that the plan-view of ACs film shows smooth surface and irregular pores formation. Figure 2(b) exhibits the surface morphology of as-prepared nano-Si@ACs composite film, which confirms the presence of nano-Si with ACs. The high resolution SEM image (shown in Fig. 2(c)) proved that the presence of nano-Si increases the surface roughness and developed uniform pores formation within ACs. The average diameter of nano-Si particles is approximately 13 nm. Figure 2(d) displays the cross-sectional micrograph of nano-Si@ACs CE, indicating the film thickness is about 12 μm. Overall, the presence of Si enables the ACs surface for fast charge transportation without any surface passivation. The obtained inter-textural porous structure assists strongly the film adhesion to FTO glass and facilitates the redox ions penetration into the film. The EDS mapping (Supplementary Fig. S2) suggests the existence of impurities in our as-prepared samples. According to literature, the existence of six metals (Na, Mg, Al, K, Ca and Fe) with Si is common in biological system^2^. These impurities might be helpful to improve the electrical conductivity of ACs and nano-Si@ACs, yielding enhanced PCE of DSSCs^2^. However, to explain the exact chemical composition of above detected impurities and their relative effect on the catalytic activity in both samples, required immense hardships. It is pertinent to mention that the presence of Mg in our samples is probably due to the magnesiothermic reduction, which is explained comprehensively in XRD results. Furthermore, the BET surface area for ACs and nano-Si@ACs are found to be ~200 m^2^g^−1^ and ~240 m^2^g^−1^, respectively.

To investigate further evidence and detail morphology of nano-Si@ACs composite, TEM and high resolution TEM (HR-TEM) had been used. Figure 2(e) shows the low magnified TEM image of nano-Si@ACs composite, which exhibits the wrinkle network of both materials *i.e.* nano-Si and ACs. This might be favorable for holes transportation by supporting each other. A HR-TEM image shown in Fig. 2(f) indicates the interface and presence of Si with ACs, represented by dotted irregular circles. Additionally, the observed HR-TEM image of ACs (as shown in Fig. 2(g)) exhibits highly porous cage-like structure and uniform pore distributions. Fast Fourier transformation (FFT) is used to determine the fringe spacing of nano-Si, which is found to be 0.313 nm as shown in Fig. 2(h). The lattice image is attributing to (111) plane of cubic Si with reference pattern (PCPDFWIN, file no. 27–1402). Figure 2(i) is the corresponding pattern of nano-Si portraying single crystalline structure.

Conventional Raman spectroscopy is non-destructive technique, widely used to illustrate the structural changes in materials. Figure 3(a & b) show the Raman analysis of nano-Si, ACs and nano-Si@ACs after annealing at 400 °C. Typically, the characteristics peaks at ~520 cm^−1^ and ~480 cm^−1^ shown in Fig. 3(a) are referred to crystalline and amorphous structures of Si, respectively^28^. As shown in Fig. 3(a), our extracted pristine nano-Si from RH exhibits two distinctive peaks at ~520 cm^−1^ and ~950 cm^−1^. The Raman result confirms that the nano-Si has single crystalline structure and match very well with literature data^1^. Figure 3(b) shows the Raman spectra for extracted ACs and nano-Si@ACs composite. As expected, two prominent peaks are observed at 1345 cm^−1^ called (D-band) and 1588 cm^−1^ (G-band), which are the characteristics of graphitic carbons^29^. The height of peaks (D/G) is indicative for relative population of sp^3^ to sp^2^ hybridized carbon species. Our calculated I_D_/I_G_ values for ACs and nano-Si@ACs are found 0.94 and 1.10 respectively, which indicates that graphitization of ACs decreases with extraction of nano-Si. Consequently, these additional active sites of nano-Si with ACs justify the improved performance of CE for DSSCs^30^.

Powder X-ray diffraction (PXRD) patterns were taken out for the crystallographic information of our extracted samples from RH. The diffraction pattern of our composite (nano-Si@ACs) shown in Fig. 3(c) depicts two characteristics broad peaks of graphitic carbon at 2θ = 26.22° and 42.21° with weak reflection for (002) and (100) planes (PCPDFWIN, file no. 75–1621) respectively. These two observed peaks for disordered carbon material indicate their turbostratic structure^8^. In addition, the XRD spectrum of our composite also confirms the pure phase of single crystalline Si, which can be indexed to reference pattern (PCPDFWIN, file no. 27–1402). Notably, at high temperature the Si nanoparticles start fusion reaction and fuses to its neighbor MgO. Thus due to the fusion reaction, part of MgO might be covered completely by Si^2^. Resultantly, during etching process, HCl has no access towards MgO, that’s why XRD detect a peak for MgO. Figure 3(d & e) show the XRD patterns for pure ACs and nano-Si that we extracted separately from RH for comparative study.

### Electrocatalytic behavior

Cyclic voltammetry (CV) was carried out to evaluate the electrocatalytic activity of our as-prepared CEs towards the reduction of I_3_^−^. Figure 4(a) shows the cyclic voltammograms curves of different CEs and their relative electrochemical data are listed in Table 1. As expected, two well-resolved pair peaks of oxidation and reduction have been observed and given by the reactions as under:


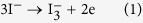



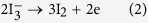



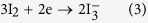



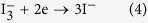


According to the literature, the two relative oxidized pair peaks are attributed to the process of oxidations *i.e.* first I^−^ oxidized to I_3_^−^ and then to I_2_ as mentioned in equations (1) & (2), respectively. In reverse reaction, first I_2_ is reduced to I_3_^−^ and next to I^−^ as depicted in equations (3) & (4)^31^. Notably, two critical parameters *i.e.* peak-to-peak splitting (E_PP_) and peak current density (I_P_) strongly influence the performance of DSSCs CE. The calculated E_PP_ values for ACs, Pt and nano-Si@ACs are found to be 0.692, 0.602 and 0.426, respectively, which shows that nano-Si@ACs has excellent catalytic activity of for I^−^/I_3_^−^ redox couple (Supplementary Fig. S3). The higher E_PP_ value (0.692) of ACs indicates its lower catalytic behavior due to the high defect ratio^32^. Furthermore, the nano-Si@ACs CE exhibits larger peaks values as compare to pristine ACs and reference Pt CEs as shown in Table 1. The higher I_P_ of our CV curves shown in (Fig. 4a) demonstrates a large electrode active surface area, which is in parallel to the result obtained in the extant literature^33^. Furthermore, the film thickness and suitable carbon contents have significant impact on the peak current density in CV curves^34^. Therefore, the excellent catalytic activity is attributed to the multifunctional synergistic catalytic effect of porous nano-Si and carbon contents (ACs &AB) in our as-prepared sample^35^. However, low power conversion efficiency and poor fill factor of individual ACs might be due to the irregular surface pore formations and inadequate redox chemistries.

Tafel-polarization measurements were performed to investigate the interfacial charge-transfer properties using the same symmetric solar cells. Figure 4(b) depicts the curves of current-density (*J*) as a function of voltage (*U*) for the redox reaction of I_3_^−^ to I^−^. The curve at low, middle and high potential region is attributed to polarization, Tafel and diffusion zone, respectively. The slope of the cathodic and anodic branches of nano-Si@ACs CE shows higher values in contrast to pristine ACs and Pt CEs, which demonstrates their superior catalytic activity^36^. The higher exchange current density (*J*_*o*_) and limiting diffusion current density (*J*_*lim*_) of the elevated slope of nano-Si@ACs CE are in parallel with EIS results in term of equation (5). The *J*_*o*_ value is inversely proportional to R_CT_ value and can be calculated by equation (5)^37^.


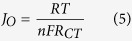


where R is the gas constant, T is the temperature, F is the Faraday’s constant, n is the number of electrons involved in the electrochemical reduction of triiodide at the electrode and R_CT_ is the charge-transfer resistance. The slightly larger *J*_*o*_ usually exhibits a superior catalytic ability to reduce I^−^/I_3_^−^ electrolyte and a higher *J*_*lim*_ indicates a larger diffusion coefficient. Additionally, the high J_SC_ value for the nano-Si@ACs CE based DSSC can also be associated to the decrease in the internal resistance components *i.e.* R_S_ and R_CT_^36^. Thus nano-Si@ACs can be a potential candidate to replace Pt as CE in DSSCs.

Electrochemical impedance spectroscopy (EIS) is a well-known technique used for further understanding of inherent differences in performance of dummy cells CEs. The obtained Nyquist plots for all CEs with equivalent circuit are illustrated in Fig. 4(c & d) and their corresponding estimated parameters are tabulated in Table 1. Typically, the intercept on real axis in Nyquist plots illustrates the series resistance (R_S_) and the diameter of left arc in high frequency range depicts the charge transfer resistance (R_CT_) called Faraday resistance and capacitance (C_μ_) of the electrode/electrolyte interface. However, the right arc in low frequency domain is assigned to the Nernst diffusion impedance (Z_N_) offered by electrolyte^38^.

As mentioned in Table 1, the estimated R_S_ value of reference Pt electrode (14.88 Ω) is slightly smaller than our extracted ACs (16.00 Ω) and nano-Si@ACs (15.90 Ω) electrodes. The obtained low R_S_ value of Pt CE reflects good bonding strength between Pt and FTO glass. However, the R_S_ value of ACs is further improved with addition of nano-Si, yielding strong contact between the film and FTO surface^39^. As shown in Table 1, the R_CT_ values for nano-Si@ACs, ACs and Pt CEs are 2.10 Ω, 3.39 Ω and 2.76 Ω, respectively. Notably, the charge transfer frequency and resistance are in inversely correlation with each other *i.e.* lower the R_CT_, higher the charge transfer frequency^40^. This proves that the higher R_CT_ value of ACs CE has lower catalytic activity among the three CEs. Once nano-Si particles are embedded in the network of ACs, the R_CT_ value of the nano-Si@ACs composite dramatically dropped to 2.10 Ω, which is much smaller than that of individual ACs (3.39 Ω) and reference Pt (2.76 Ω). Obviously, the Z_N_ values of the three as-prepared samples are in an order of nano-Si@ACs (27.9 Ω) < Pt (29.1 Ω) < ACs (30 Ω). The smaller Z_N_ value of the nano-Si@ACs indicates a faster diffusion velocity of the redox couple in the electrolyte. The improved electrocatalytic activity confirms that nano-Si@ACs is superior electrocatalyst, attributed to the synergistic coupling effect between ACs and nano-Si^35^.

### Photovoltaic performance

We now turn to decipher the role of nano-Si with ACs extracted from RH; *J-V* (current density *versus* voltage) performances were studied. The photovoltaic parameters such as open-circuit voltage (V_OC_), short-circuit current density (J_SC_) and fill factor (FF) with their relative efficiencies (*η*) of various CEs are summarized in Table 2. Figure 5(a & b) show the *J-V* curves of various DSSCs under standard illumination and dark conditions. Notably, three necessary factors *i.e.* electron transport, catalytic active sites and diffusion of electrolyte could affect the reduction reaction (I_3_^−^/I^−^) at interface of electrocatalyst^41^. Therefore, the outstanding catalytic activity must be suitable combination of above three random factors.

Owing to above specified parameters (such as V_OC_, J_SC_ and FF) nano-Si@ACs offers superior PCE (8.01%) in contrast to pristine ACs (6.57%) and conventional Pt (7.20%), which are explained sequentially: (i) Short-circuit photocurrent: As shown in Fig. 5(a) ACs (15.01 mA cm^−2^) and nano-Si@ACs (15.50 mA cm^−2^) depicts remarkable J_SC_ values, which is comparable to reference Pt (15.20 mA cm^−2^). The exceptional J_SC_ values might be due to addition of acetylene black into both ACs and nano-Si@ACs samples. However, the J_SC_ value of ACs is further improved by extraction of nano-Si with ACs surface; (ii) Open-circuit potential: Under same experimental conditions, nano-Si@ACs CE depicts higher V_OC_ (0.76 V) value than that of ACs (0.73 V) and conventional Pt (0.74 V) CEs of DSSCs. Indeed, V_OC_ is the difference of Fermi level of semiconducting material and potential energy of redox potential in electrolyte, which is influenced by instinct material properties of the CEs^42^. Therefore, higher V_OC_ for nano-Si@ACs is the result of its higher J_SC_ value; and (iii) Fill factor: In fact, FF is closely associated to charge transfer resistance at CE of DSSCs. Therefore, the higher FF value of nano-Si@ACs is the result of available chemical defects for fast redox reaction at CE/electrolyte interface. The improved FF is due to combination of suitable amounts of carbon contents and the presence of nano size Si, describes high specific surface area. Furthermore, the presence of nano-Si with ACs and AB minimizes the charge recombination as well as provides lower charge transfer resistance at film surface, thus accounts for enhanced J_SC_ value.

### Stability tests

In order to observe the preliminary chemical stability of various CEs, we performed dark *J-V* tests as shown in Fig. 5(b). As compare to ACs and Pt, the nano-Si@ACs based DSSC yields a smaller dark current, which indicates that the reduction of I_3_^−^ on the electrolyte/CE interface is quite efficient and the reduction of I_3_^−^ on the TiO_2_/electrolyte interface is retarded. Obviously, there are no abnormal differences perceived in the dark current, representing that the nano-Si@ACs CE would not react with the electrolyte containing I_3_^−^/I^−^ redox couple^43^.

The evaluation of the long-term practical stability of nano-Si@ACs CE in DSSC is the critical concern for its applications. As shown by viewing the plots displayed in Fig. 6(a–d), the photovoltaic parameters (J_SC_, V_OC_, FF and PCE) of dummy cell varies with aging time. During testing time, it is observed that both V_OC_ and FF slightly increased with time as compared with fresh one. However, the values of J_SC_ decreased continuously with passage of time. The fall in minor J_SC_ can compensate for increase in both V_OC_ and FF, resultantly the device preserves its initial day performance^44^. The time stability test exhibits the robustness of nano-Si@ACs CE in the I^−^/I_3_^−^ redox electrolyte. All results confirm that the extraction of nano-Si with ACs from RH can greatly improve the performance of CE.

Usually, a multi-cycle successive CV scanning is adopted to judge the electrochemical stability of DSSCs CE materials. The 1^st^ and 30^th^ successive CV cycles (at a scan rate of 10 mV S^−1^) of two-month-aged porous nano-Si@ACs electrode are shown in Fig. 6(e). After 30 cycles of continuous scanning, the peak current density and curve shape are unchanged, which demonstrate good electrochemical stability of nano-Si@ACs electrode in the I_3_^−^/I^−^ electrolyte system. This indicates that no specific interaction occurred between nano-Si@ACs CE and I ^−^/I_3_^−^ couples. The excellent electrochemical stability can be attributed to the combine porous structure of ACs and AB^45^ with nano-Si particles, which shows that nano-Si@ACs is useful candidate for DSSCs CE.

EIS can also be employed to evaluate the chemical stability of CE materials^46^. Figure 6(f) shows the repeated EIS tests of nano-Si@ACs after one-cycle successive CV scanning. For nano-Si@ACs electrode, there is almost no change of R_CT_ after 10 cycles of scanning. The unchanged R_CT_ indicates that our as-prepared nano-Si@ACs composite has excellent electro-chemical stability against potential cycling. Furthermore, there is negligible change occurred in R_S_ and Z_N_, indicates that the potential cycling hardly influenced the series ohmic resistance and the mass transport in the redox electrolyte^47^.

## Discussion

Pt is an attractive candidate for high performance CE of DSSCs due to its tremendous conductivity and superb electrocatalytic properties. Beside their large scale applications, Pt electrode has certain shortcomings: (i) highly expensive and limited supply^48^; (ii) weak corrosion resistance for I-based electrolyte^49^; (iii) as a CE material of DSSCs, Pt is not useful for the I-free redox couple (such as T_2_/T^−^, Co(III)/(II) and polysulfide) electrolyte reaction^45^; (iv) it can be toxic by air components^50^; and (v) couldn’t match with emerging new concepts of solar cell constituents like new dyes, electrolytes and anode materials^45^ *etc*. Consequently, it is quite necessary to find credible alternative Pt-free CE materials for low cost DSSCs. In this work, while aiming useful exploitation of RH in CE of DSSCs, we extracted nano-Si with ACs from RH simultaneously. The acquired nano-Si@ACs composite possesses superior electrical conductivity and catalytic activity in contrast to pristine ACs. In addition, DSSC assembled with porous structure of nano-Si@ACs CE has shown comparable PCE to that of conventional Pt CE. Unlike other recycled procedures for extraction of nano-Si or ACs from RH for CEs of DSSCs, our developed process for nano-Si@ACs is least energy exhaustive. These divergent characteristics pave the way for making low-cost and highly efficient CE for DSSCs.

In summary, we explored the extraction of nano-Si@ACs from RH and utilized as CE material for DSSCs. The morphology, composition and inter-textural structure of as-prepared composite are characterized by various techniques. Our nano-Si@ACs CE based DSSC showed much higher light-to-electricity PCE than pristine ACs CE. The DSSC with nano-Si@ACs CE has solar to electrical PCE of 8.01%, which is comparable to a reference Pt-based CE DSSC (7.20%). Furthermore, the extraction of nano-Si with ACs from RH enhanced the electrocatalytic activity of ACs due to their multifunctional synergistic electrocatalytic effect and also delivered suitable graphitization degree. Based on the obtained results, it is suggested that nano-Si@ACs can ultimately perform better as compare to pristine ACs. Our results exposed new possibilities for utilization of suitable biomass derived nano-Si and activated carbons as promising low-cost alternatives to the costly Pt in the CE of DSSCs.

## Methods

### Extraction of nano-silicon with activated carbons

**Figure 1:**
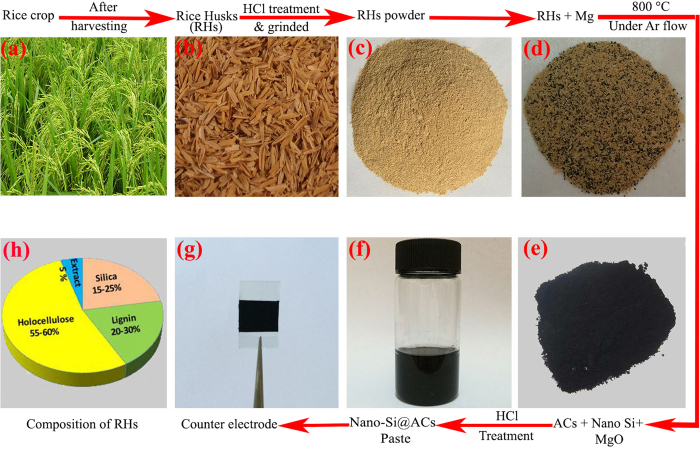
Flow chart for extraction of nano-Si@ACs from rice plants. (**a**) Photograph of rice plants grown on farm. Optical images of (**b**) raw RH obtained after threshing, (**c**) RH after grinding, (**d**) RH/Mg mixture, (**e**) after thermal decomposition and magnesiothermic reduction processes, (**f**) obtained nano-Si@ACs after acids treatment, (**g**) as-prepared CE and (**h**) circular chart representing the composition of RH. (Photographed by Waqar Ahmad).

**Figure 2:**
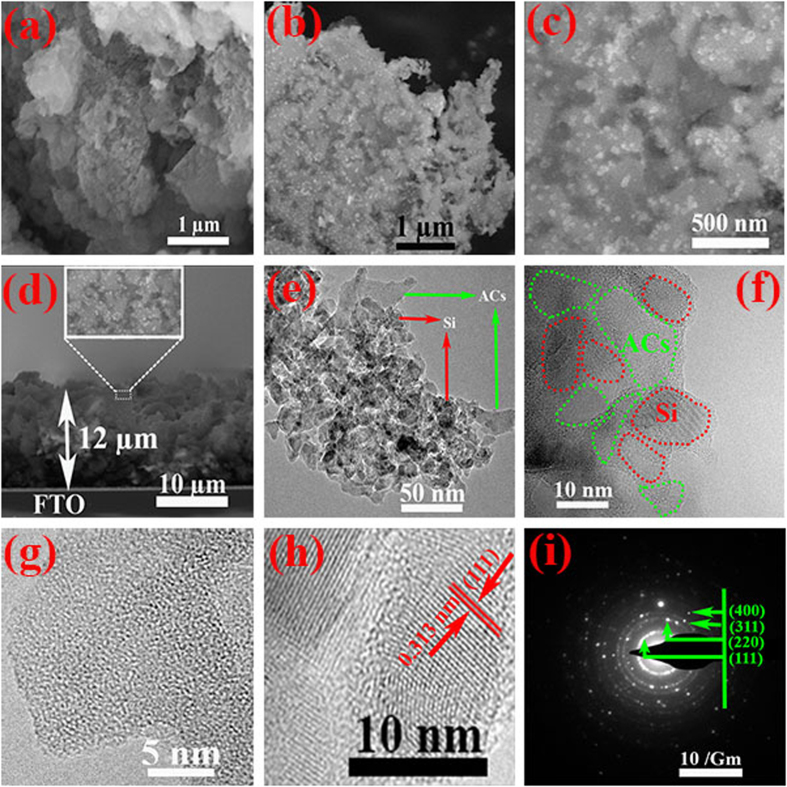
(**a,b**) FE-SEM micrographs of ACs and nano-Si@ACs, respectively; (**c,d**) High resolution surface and cross-sectional SEM images of as-prepared nano-Si@ACs film, (**e,f**) TEM images with green and red arrows and dotted irregular circles depicts ACs and nano-Si of nano-Si@ACs composite, respectively. (**g,h**) Show the HR-TEM images of ACs and nano-Si, respectively. (**i**) FFT pattern of nano-Si extracted from RH simultaneously. All surface images of the samples were taken without addition of acetylene black (AB).

**Figure 3:**
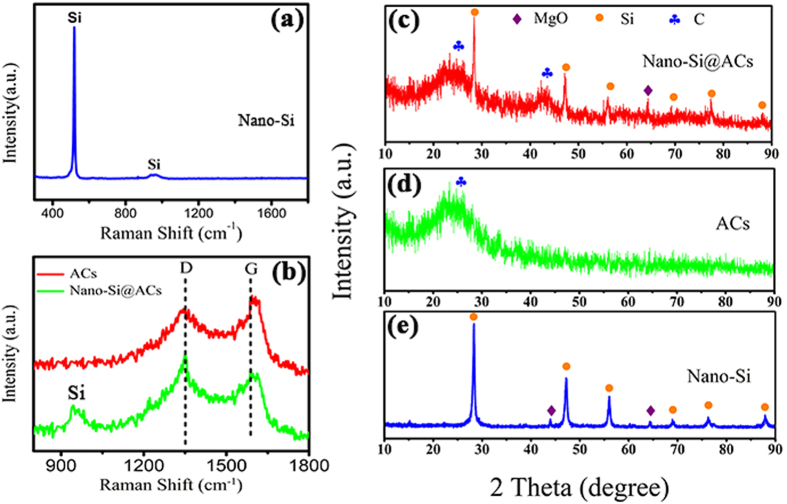
(**a,b**) Raman spectra and (**c**–**e**) PXRD of individual extracted nano-Si, ACs and composite of nano-Si@ACs (before addition of AB), respectively.

**Figure 4:**
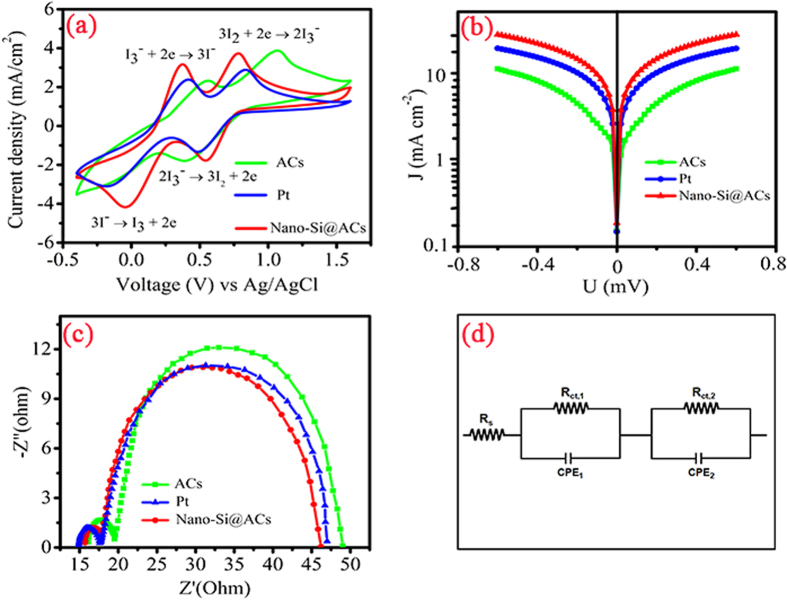
(**a**) Cyclic voltammetry (CV), (**b**) Tafel polarization and (**c**) Electrochemical impedance spectroscopy (EIS) plots of ACs, nano-Si@ACs (after addition of AB) and Pt CEs of symmetrical dummy cells with (**d**) equivalent circuit diagram.

**Figure 5:**
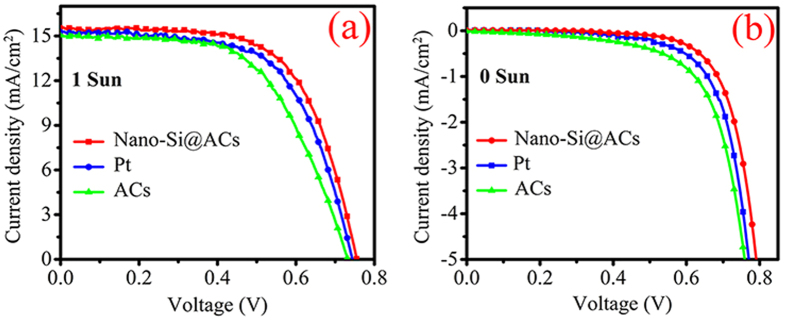
The current-density voltage (*J-V*) curves of DSSCs with ACs, nano-Si@ACs (after addition of AB) and Pt CEs under (**a**) 1 sun illumination (100 mW cm^−2^) and (**b**) 0 sun illumination condition.

**Figure 6:**
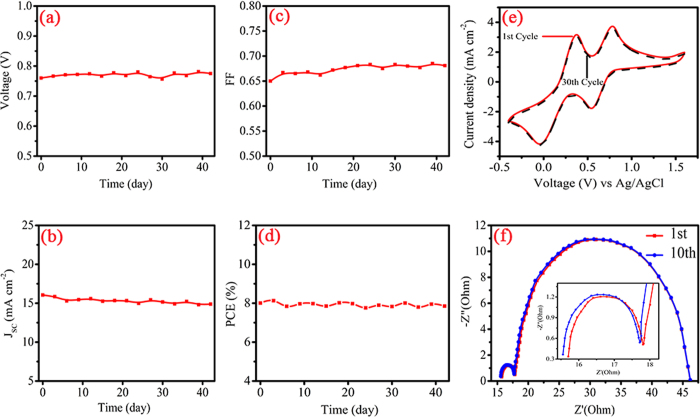
(**a–d**) Variations of photovoltaic parameters with time at room temperature for nano-Si@ACs based dummy cell. (**e**) 1^st^ and 30^th^ successive CVs of aged nano-Si@ACs CE with I^−^/I_3_^−^ redox couples and (**f**) EIS stability test of symmetrical dummy cell with nano-Si@ACs electrode. The cell was first subjected to CV scanning with a scan rate of 100 mV s^−1^ (from 0 V → 1 V → −1 V → 0 V), followed by 20 s relaxation at 0 V, and then EIS measurement at 0 V from 0.1 Hz to 0.1 MHz was performed. This sequential electrochemical test was repeated 10 times.

The synthetic procedure adopted for extraction of nano-Si@ACs is illustrated in Fig. 1. In brief, a certain amount of commercially available RH was refluxed thoroughly with 10% HCl solution to remove the inside metal ions. The leached RH was washed with distilled water several times and dried at 100 °C for a period of 12 h. After initial drying, 0.8 g RH was carefully ground and mixed with (0.23 g) Mg powder. To produce nano-Si with ACs, the resulting mixed powder was treated in tube furnace at 850 °C for 2 h with ramping rate of 5–7 °C min^−1^ under protection of argon flow^29^. During this process, the RH and Mg were converted into mixture of nano-Si@ACs, MgO and Mg_2_Si. The obtained composite was soaked in 1 M HCl solution (using molar ratio of HCl: H_2_O: EtOH = 0.66: 4.72: 8.88) at room temperature^29^ to remove MgO and Mg_2_Si and then reacted with 5% HF solution for 1 h to make sure that any residual or newly formed silica was washed out. Finally, the wet precipitates (nano-Si@ACs) were dried overnight at 70 °C in vacuum oven.

### Preparation of counter electrode

0.6 g of the obtained nano-Si@ACs was mixed with 0.4 g (40 wt%) acetylene black (our previously published procedure)^51^ and some ethanol in 25 mL beaker under stirring at room temperature. To attain fine dispersion, 0.2 mL acetic acid, 0.50 g ethyl cellulose and 3.0 g terpineol anhydrous were dispersed in the above solution. After vigorous agitation, the nano-Si@ACs paste was coated onto patterned conducting layer of FTO glass by means of Dr. Blade method. The resultant electrodes were sintered gradually at 400 °C for 1 h to obtain 3-D network structure of composite using muffle furnace. For comparative study, we also extracts ACs^8^ separately and then mixed with AB (0.4 g, 40 wt%), followed by above experimental approach. The standard Pt CE was prepared as stated by literature^52^.

### Fabrication of DSSCs

We synthesized the mesoporous *n*-TiO_2_ colloidal paste according to ref. 53. A commercial P25 (Degussa, Germany) powder with mean particle size ~20 nm was used. In brief, for compact layer of TiO_2_, the conductive glassy slides were immersed into 40 mM TiCl_4_ aqueous solution for 0.5 h at 70 °C. Subsequently, the mesoporous *n*-TiO_2_ suspension was layered onto the treated FTO glasses using one layer of magic scotch tape (with thickness of 50 μm) via Dr. Blade method and dried at 130 °C for 0.5 h. To thicken *n*-TiO_2_ films, the coating process was repeated and annealed at 500 °C for 1 h. To avoid any recombination of e^−^/h^+^ pairs, the resultant *n*-TiO_2_ photoelectrodes (PEs) were treated again with 40 mM TiCl_4_ solution. Later, the substrates were washed thoroughly with ethanol and re-calcined at 520 °C for 0.5 h. Then the as-prepared samples were allowed to cool to 80 °C and immersed into 0.5 mM of N719 dye in acetonitrile/tert-butanol solution (V:V/1:1) for 20 h. Finally, the DSSC was assembled by sandwiching dye sensitized PE, CE and electrolyte solution. The electrolyte was composed of 0.03 M I_2_, 0.05 M LiI, 0.6 M PMII, 0.1 M GuSCN and 0.5 M 4-TBP in acetonitrile and valeronitrile (V:V/85:15) as solvents. The effective area of the solar cell was 0.15 cm^2^ without mask.

### Characterization

Thermal gravimetric analysis (TGA) measurements were carried out using Perkin-Elmer Diamond (Pyris1 TGA). X-ray diffraction (XRD, PANalytical B.V. Netherland) system using Cu-Kα radiation wavelength (1.54060 Å) and Brunauer-Emmett-Teller method (BET, ASAP 2020 V4.00 USA) were used to characterize the crystal structure and specific surface area of as-prepared samples. Field emission scanning electron microscopy (FE-SEM, FEI NOVA NanoSEM 450), Transmission electron microscopy (TEM, FEI Tecnai G^2^ 20 UTwin) and Raman spectroscopy (HORIBA Jobin Yvon UV-VIS-NIR LabRAM, 532 nm) were used to investigate the nano-composite structures and cross-sectional morphologies of the samples. Energy-dispersive X-ray spectroscopy (EDS) equipped with FE-SEM was carried out to analyze the various elements in the prepared films.

### Electrochemical measurements of DSSCs

The produced current-voltage curves of dummy cells were measured under standard illumination coming from a solar simulator (Newport, USA) with AM 1.5 G (100 mW cm^−2^). Electrochemical impedance spectroscopy (EIS) and Tafel polarization measurements were carried out by using an Auto-Lab electrochemical workstation (model AUT84315, the Netherlands) and electrolyte was the same as the electrolyte of DSSCs. In EIS measurement, the recorded frequency ranged from 0.1 Hz to 0.1 MHz under 1 sun illumination. A potentiostat (CHI660C instrument) in a three electrode system was used to measure cyclic voltammetric scannings (CV) at a scan rate of 50 mVs^−1^. The electrolyte solution was composed of 1 mM I_2_, 10 mM LiI, and 100 mM LiClO_4_.3H_2_O in acetonitrile. A Pt foil and Ag/AgCl were used as a counter electrode and reference electrode, respectively.

## Additional Information

**How to cite this article**: Ahmad, W. *et al*. Extraction of nano-silicon with activated carbons simultaneously from rice husk and their synergistic catalytic effect in counter electrodes of dye-sensitized solar cells. *Sci. Rep.* **6**, 39314; doi: 10.1038/srep39314 (2016).

**Publisher's note:** Springer Nature remains neutral with regard to jurisdictional claims in published maps and institutional affiliations.

## Supplementary Material

Supplementary Information

## Figures and Tables

**Table 1:** EIS and CV parameters of various CEs of DSSCs.

Counter electrode	R_S_ (Ω)	R_CT_ (Ω)	Z_N_ (Ω)	E_PP_	I_pa_ (mAcm^−2^)	I_pc_ (mAcm^−2^)
ACs	16.00	3.39	30.0	0.692	2.327	−2.824
Nano-Si@ACs	15.90	2.10	27.9	0.426	3.165	−4.172
Pt	14.88	2.76	29.1	0.602	2.386	−3.103

**Table 2:** Photovoltaic performance list of DSSCs with various CEs under AM 1.5 G light illumination.

Counter electrode	V_OC_ (V)	J_SC_ (mA cm^−2^)	FF	PCE (%)	∆PCE
ACs	0.73	15.01	0.60	6.57	—
Nano-Si@ACs	0.76	15.50	0.67	8.01	22%
Pt	0.74	15.20	0.64	7.20	—

## References

[b1] Jung D. S., Ryou M.-H., Sung Y. J., Park S. B. & Choi J. W. Recycling rice husks for high-capacity lithium battery anodes. Proc. Nat. Acad. Sci. 110, 12229–12234 (2013). 23836636 10.1073/pnas.1305025110 PMC3725048

[b2] Liu N., Huo K., McDowell M. T., Zhao J. & Cui Y. Rice husks as a sustainable source of nanostructured silicon for high performance Li-ion battery anodes. Sci. Rep. 3, 1919–1925 (2013). 23715238 10.1038/srep01919 PMC3665957

[b3] Wang L., Schnepp Z. & Titirici M. M. Rice husk-derived carbon anodes for lithium ion batteries. J. Mater. Chem. A 1, 5269–5273 (2013).

[b4] Navarro-Suárez A. M. . Nanoporous carbons from natural lignin: study of structural–textural properties and application to organic-based supercapacitors. RSC Adv. 4, 48336–48343 (2014).

[b5] Chen Y. . Dye removal of activated carbons prepared from NaOH-pretreated rice husks by low-temperature solution-processed carbonization and H_3_PO_4_ activation. Bioresour. Technol. 144, 401–409 (2013). 23892148 10.1016/j.biortech.2013.07.002

[b6] Chen H. . Extraction of lignocellulose and synthesis of porous silica nanoparticles from rice husks: a comprehensive utilization of rice husk biomass. ACS Sustainable Chem. Eng. 1, 254–259 (2012).

[b7] Guo Y. . Performance of electrical double layer capacitors with porous carbons derived from rice husk. Mater. Chem. Phy. 80, 704–709 (2003).

[b8] Wang G., Wang D., Kuang S., Xing W. & Zhuo S. Hierarchical porous carbon derived from rice husk as a low-cost counter electrode of dye-sensitized solar cells. Renew. Energy 63, 708–714 (2014).

[b9] Yun S., Hagfeldt A. & Ma T. Pt-Free Counter Electrode for Dye-Sensitized Solar Cells with High Efficiency. Adv. Mater. 26, 6210–6237 (2014). 25080873 10.1002/adma.201402056

[b10] Yun S., Liu Y., Zhang T. & Ahmad S. Recent advances in alternative counter electrode materials for Co-mediated dye-sensitized solar cells. Nanoscale 7, 11877–11893 (2015). 26132719 10.1039/c5nr02433a

[b11] Hao F. . Recent advances in alternative cathode materials for iodine-free dye-sensitized solar cells. Energy Environ. Sci. 6, 2003–2019 (2013).

[b12] Zhang J. . A 12%-Efficient Upgraded Metallurgical Grade Silicon–Organic Heterojunction Solar Cell Achieved by a Self-Purifying Process. ACS Nano 8, 11369–11376 (2014). 25365397 10.1021/nn504279d

[b13] Ahmad S., Guillen E., Kavan L., Gratzel M. & Nazeeruddin M. K. Metal free sensitizer and catalyst for dye sensitized solar cells. Energy Environ. Sci. 6, 3439–3466 (2013).

[b14] Neergaard Waltenburg H. & Yates J. Surface chemistry of silicon. Chem. Rev. 95, 1589–1673 (1995).

[b15] Cohn A. P. . All Silicon Electrode Photocapacitor for Integrated Energy Storage and Conversion. Nano Lett. 15, 2727–2731 (2015). 25806838 10.1021/acs.nanolett.5b00563

[b16] Yang R., Buonassisi T. & Gleason K. K. Organic vapor passivation of silicon at room temperature. Adv. Mater. 25, 2078–2083 (2013). 23355317 10.1002/adma.201204382

[b17] Mariotti D., Mitra S. & Švrček V. Surface-engineered silicon nanocrystals. Nanoscale 5, 1385–1398 (2013). 23334154 10.1039/c2nr33170e

[b18] Sanfins E. . Carbon black nanoparticles impair acetylation of aromatic amine carcinogens through inactivation of arylamine N-acetyltransferase enzymes. ACS Nano 5, 4504–4511 (2011). 21526848 10.1021/nn103534d

[b19] Yao Y. & Wu F. Naturally derived nanostructured materials from biomass for rechargeable lithium/sodium batteries. Nano Energy 17, 91–103 (2015).

[b20] Wong D. P. . Binder-free rice husk-based silicon–graphene composite as energy efficient Li-ion battery anodes. J. Mater. Chem. A 2, 13437–13441 (2014).

[b21] Liu Y. . A sustainable route for the preparation of activated carbon and silica from rice husk ash. J. Hazard. Mater. 186, 1314–1319 (2011). 21194835 10.1016/j.jhazmat.2010.12.007

[b22] Liu Y. . Simultaneous preparation of silica and activated carbon from rice husk ash. J. Cleaner Prod. 32, 204–209 (2012).

[b23] Bao Z. . Chemical reduction of three-dimensional silica micro-assemblies into microporous silicon replicas. Nature 446, 172–175 (2007). 17344850 10.1038/nature05570

[b24] Kalderis D., Bethanis S., Paraskeva P. & Diamadopoulos E. Production of activated carbon from bagasse and rice husk by a single-stage chemical activation method at low retention times. Bioresour. Technol. 99, 6809–6816 (2008). 18364254 10.1016/j.biortech.2008.01.041

[b25] Williams P. T. & Besler S. The pyrolysis of rice husks in a thermogravimetric analyser and static batch reactor. Fuel 72, 151–159 (1993).

[b26] Figueiredo J. L., Pereira M. F. R., Freitas M. M. A. & Órfão J. J. M. Modification of the surface chemistry of activated carbons. Carbon 37, 1379–1389 (1999).

[b27] Le Van K. & Luong Thi T. T. Activated carbon derived from rice husk by NaOH activation and its application in supercapacitor. Prog. Nat. Sci. Mater. Int. 24, 191–198 (2014).

[b28] Fu K. . Aligned Carbon Nanotube‐Silicon Sheets: A Novel Nano‐architecture for Flexible Lithium Ion Battery Electrodes. Adv. Mater. 25, 5109–5114 (2013). 23907770 10.1002/adma.201301920

[b29] Zhang L. . Highly graphitized nitrogen-doped porous carbon nanopolyhedra derived from ZIF-8 nanocrystals as efficient electrocatalysts for oxygen reduction reactions. Nanoscale 6, 6590–6602 (2014). 24806824 10.1039/c4nr00348a

[b30] Hsieh C.-T., Yang B.-H. & Lin J.-Y. One-and two-dimensional carbon nanomaterials as counter electrodes for dye-sensitized solar cells. Carbon 49, 3092–3097 (2011).

[b31] Fang H., Yu C., Ma T. & Qiu J. Boron-doped graphene as a high-efficiency counter electrode for dye-sensitized solar cells. Chem. Commun. 50, 3328–3330 (2014). 10.1039/c3cc48258h 24535331

[b32] Punckt C., Pope M. A., Liu J., Lin Y. & Aksay I. A. Electrochemical performance of graphene as effected by electrode porosity and graphene functionalization. Electroanal. 22, 2834–2841 (2010).

[b33] Wu M., Lin X., Wang T., Qiu J. & Ma T. Low-cost dye-sensitized solar cell based on nine kinds of carbon counter electrodes. Energy Environ. Sci. 4, 2308–2315 (2011).

[b34] Yue G. . High performance platinum-free counter electrode of molybdenum sulfide-carbon used in dye-sensitized solar cells. J. Mater. Chem. A 1, 1495–1501 (2013).

[b35] Erwin W. R. . Engineered Porous Silicon Counter Electrodes for High Efficiency Dye-Sensitized Solar Cells. ACS Appl. Mater. Interfaces 6, 9904–9910 (2014). 24884149 10.1021/am503257e

[b36] Chen S. . *In-Situ* and Green Method To Prepare Pt-Free Cu_2_ZnSnS_4_ (CZTS) Counter Electrodes for Efficient and Low Cost Dye-Sensitized Solar Cells. ACS Sustainable Chem. Eng. 3, 2652–2659 (2015).

[b37] Ju M. J. . N-Doped Graphene Nanoplatelets as Superior Metal-Free Counter Electrodes for Organic Dye-Sensitized Solar Cells. ACS Nano 7, 5243–5250 (2013). 23656316 10.1021/nn4009774

[b38] Satoh N. & Han L. Chemical input and I-V output: stepwise chemical information processing in dye-sensitized solar cells. Phys. Chem. Chem. Phys. 14, 16014–16022 (2012). 23104104 10.1039/c2cp43460a

[b39] Dong P. . Vertically aligned single-walled carbon nanotubes as low-cost and high electrocatalytic counter electrode for dye-sensitized solar cells. ACS Appl. Mater. Interfaces 3, 3157–3161 (2011). 21770421 10.1021/am200659y

[b40] Yu C. . Graphene-mediated highly-dispersed MoS_2_ nanosheets with enhanced triiodide reduction activity for dye-sensitized solar cells. Carbon 100, 474–483 (2016).

[b41] He J., Pringle J. M. & Cheng Y.-B. Titanium carbide and titanium nitride-based nanocomposites as efficient catalysts for the Co^2+^/Co^3+^ redox couple in dye-sensitized solar cells. J. Phys. Chem. C 118, 16818–16824 (2014).

[b42] Hsu S.-H. . Platinum-free counter electrode comprised of metal-organic-framework (MOF)-derived cobalt sulfide nanoparticles for efficient dye-sensitized solar cells (DSSCs). Sci. Rep. 4, 6983–6988 (2014). 25382139 10.1038/srep06983 PMC4225550

[b43] Yun S., Pu H., Chen J., Hagfeldt A. & Ma T. Enhanced Performance of Supported HfO_2_ Counter Electrodes for Redox Couples Used in Dye-Sensitized Solar Cells. ChemSusChem 7, 442–450 (2014). 24399514 10.1002/cssc.201301140

[b44] Lee W. J., Ramasamy E., Lee D. Y. & Song J. S. Efficient dye-sensitized solar cells with catalytic multiwall carbon nanotube counter electrodes. ACS Appl. Mater. Interfaces 1, 1145–1149 (2009). 20355903 10.1021/am800249k

[b45] Yun S., Lund P. D. & Hinsch A. Stability assessment of alternative platinum free counter electrodes for dye-sensitized solar cells. Energy Environ. Sci. 8, 3495–3514 (2015).

[b46] Kavan L., Yum J. H. & Grätzel M. Optically transparent cathode for dye-sensitized solar cells based on graphene nanoplatelets. ACS Nano 5, 165–172 (2010). 21126092 10.1021/nn102353h

[b47] Gong F., Wang H., Xu X., Zhou G. & Wang Z.-S. *In situ* growth of Co_0.85_Se and Ni_0.85_Se on conductive substrates as high-performance counter electrodes for dye-sensitized solar cells. J. Am. Chem. Soc. 134, 10953–10958 (2012). 22713119 10.1021/ja303034w

[b48] Al-bahrani M. R. . Layer-by-layer deposition of CNT^−^ and CNT^+^ hybrid films for platinum free counters electrodes of dye-sensitized-solar-cells. RSC Adv. 5, 95551–95557 (2015).

[b49] Ahmad W. . Formation of short three dimensional porous assemblies of super hydrophobic acetylene black intertwined by copper oxide nanorods for a robust counter electrode of DSSCs. RSC Adv. 5, 35635–35642 (2015).

[b50] Hou Y. . Rational screening low-cost counter electrodes for dye-sensitized solar cells. Nat. Commun. 4, 1583–1590 (2013). 23481391 10.1038/ncomms2547

[b51] Ahmad W. . P-type NiO nanoparticles enhanced acetylene black as efficient counter electrode for dye-sensitized solar cells. Mater. Res. Bull. 67, 185–190 (2015).

[b52] Ahn S. H. & Manthiram A. Edge‐Oriented Tungsten Disulfide Catalyst Produced from Mesoporous WO_3_ for Highly Efficient Dye‐Sensitized Solar Cells. Adv. Energy Mater. 6, 1501814–1501820 (2016).

[b53] Yi L. . One dimensional CuInS_2_-ZnS heterostructured nanomaterials as low-cost and high-performance counter electrodes of dye-sensitized solar cells. Energy Environ. Sci. 6, 835–840 (2013).

