# Supplementary material for: Extraction of nano-silicon with activated carbons simultaneously from rice husk and their synergistic catalytic effect in counter electrodes of dye-sensitized solar cells
Source: Sci Rep. 2016 Dec 21;6:39314. doi: 10.1038/srep39314 (PMC5175195; doi:10.1038/srep39314)
Supplement: Supplementary Information [file srep39314-s1.pdf]

Supplementary Informations

Extraction of nano-silicon with activated carbons simultaneously from rice husk and their synergistic catalytic effect in counter electrodes of dye-sensitized solar cells

Waqar Ahmad[a], Majid Raissan Al bahrani[a], Zhichun Yang[a], Jahangeer Khan[a], Wenkui Jing[a], Fan Jiang[a], Liang Chu[b], Nishuang Liu[a], Luying Li[a] and Yihua Gao[*ac]

[a]Center for Nanoscale Characterization & Devices (CNCD), Wuhan National Laboratory for Optoelectronics (WNLO) & School of Physics, Huazhong University of Science and Technology (HUST), Luoyu Road 1037, Wuhan 430074, P. R. China

[b]Center of Advanced Functional Ceramics (CAFC), Nanjing University of Posts and Telecommunications (NUPT), Nanjing 210046, P. R. China

[c]Hubei Collaborative Innovation Center for Advanced Organic Chemical Materials, 368 Youyi Avenue, Wuhan 430062, P. R. China

Correspondence and requests for materials should be addressed to Y.G. (*email: **gaoyihua@hust.edu.cn**)

Materials and Reagents

All chemical and reagents were of analytical grade and used as received from suppliers. Commercially available natural RH were obtained from local market and thoroughly washed with distilled water and HCl solution. Magnesium oxide (MgO), ethanol (C_2H_6O), terpineol anhydrous ($C_{10}H_{18}O$), titanium tetrachloride ($TiCl_4$), isopropyl alcohol (C_3H_8O), acetic acid (HAc), hydrochloric acid (HCl) and hydrofluoric acid (HF) were purchased from Sinopharm Chemical Reagent Co., Ltd. Acetylene (99%) was obtained from Hubei Chuchengwei Chemical

Co., Ltd. N719 (cis–bis (isothiocyanato) bis(2,2'-bipyridyl-4,4'-dicarboxylato) ruthenium(II) bis-tetrabutylammonium) was supplied by Solaronix. Fluorine doped tin oxide glass (FTO, 7 Ω cm^{-2}) was purchased from Nippon Sheet Glass Co. Ltd. and washed ultrasonically with laundry soap, acetone, Milli-Q ultra-pure water (18.2 MΩ-cm) and ethanol for 15 minutes, respectively. Lithium iodide (LiI, 99.999%), iodine (I_2, 99.99%), $TiCl_4$. $3H_2O$ (99.99%), 1-methyl-3-propylimidazolium iodide (PMII, 98%), 4-tert-butylpyridine (4-TBP, 96%), tert-butyl alcohol (99.5 %) and nickel oxide (NiO, 99.5%, 30 nm beads) were obtained from Aladdin. Acetonitrile (99.8%) and valeronitrile (99%) were bought from Alfa Aesar.

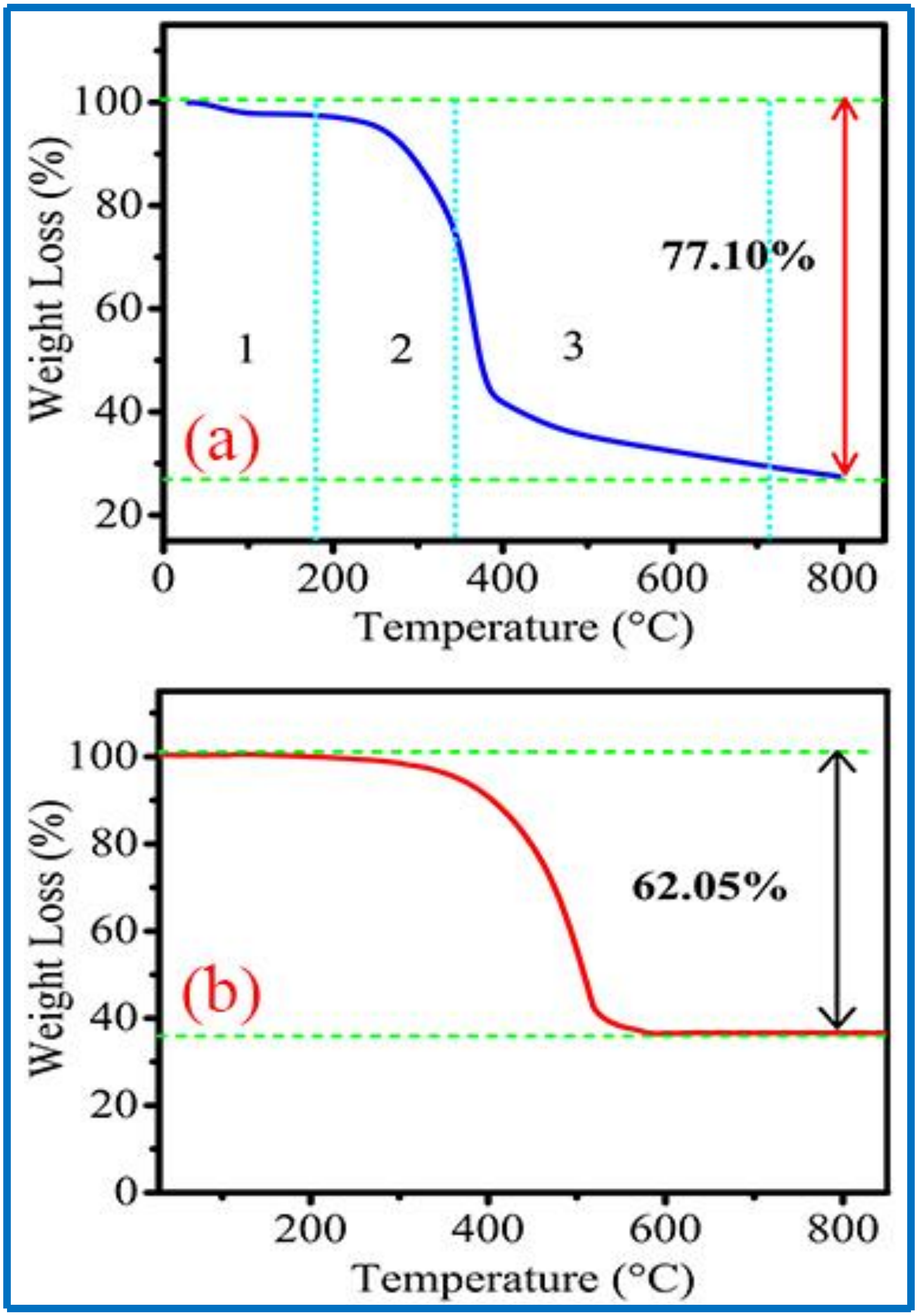

Figure S1. TGA thermograms of (a) RH after acid leaching and (b) extracted composite of nano-Si@ACs.

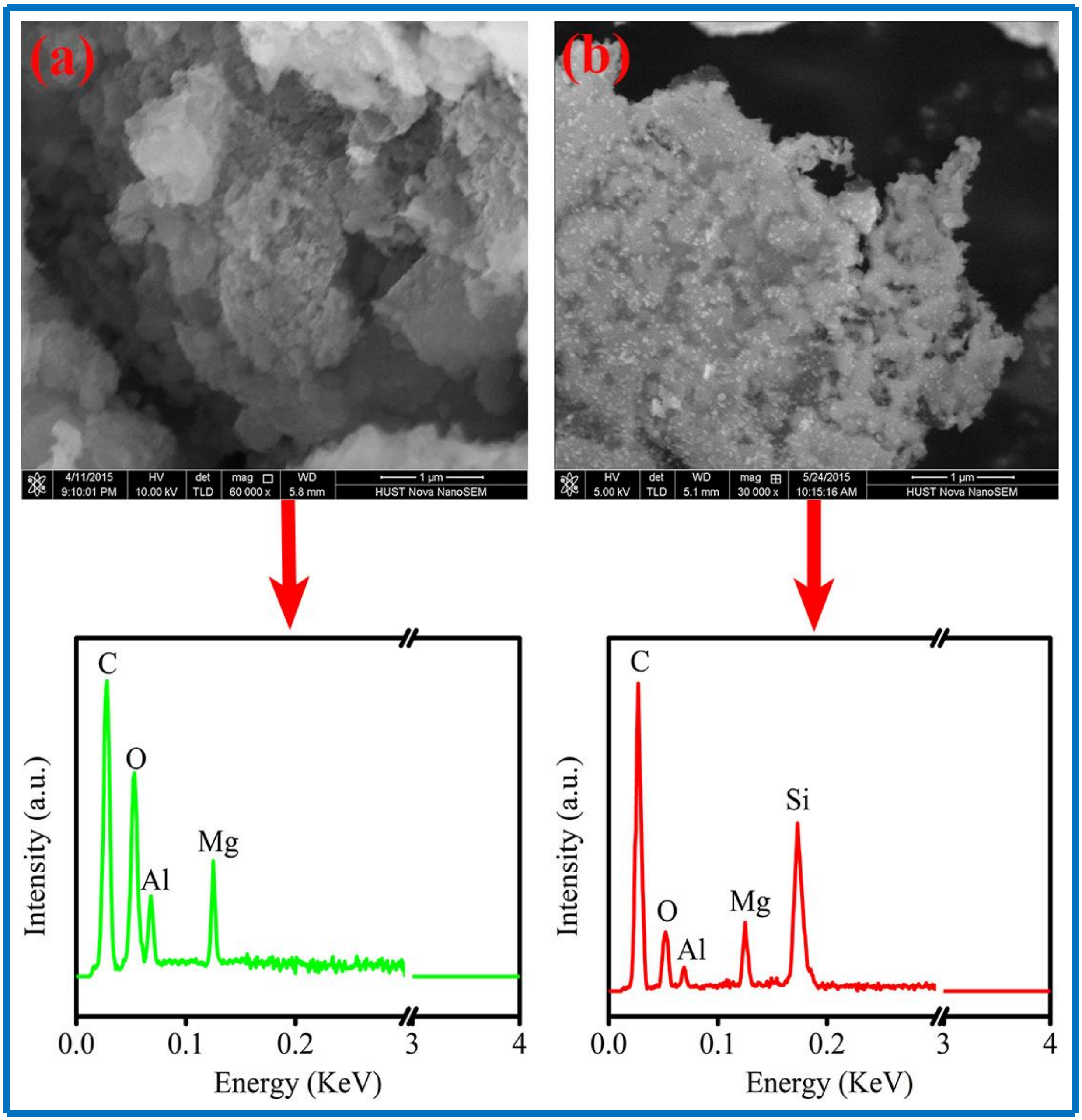

Figure S2. EDS maping of extracted (a) ACs and (b) nano-Si@ACs from RH.

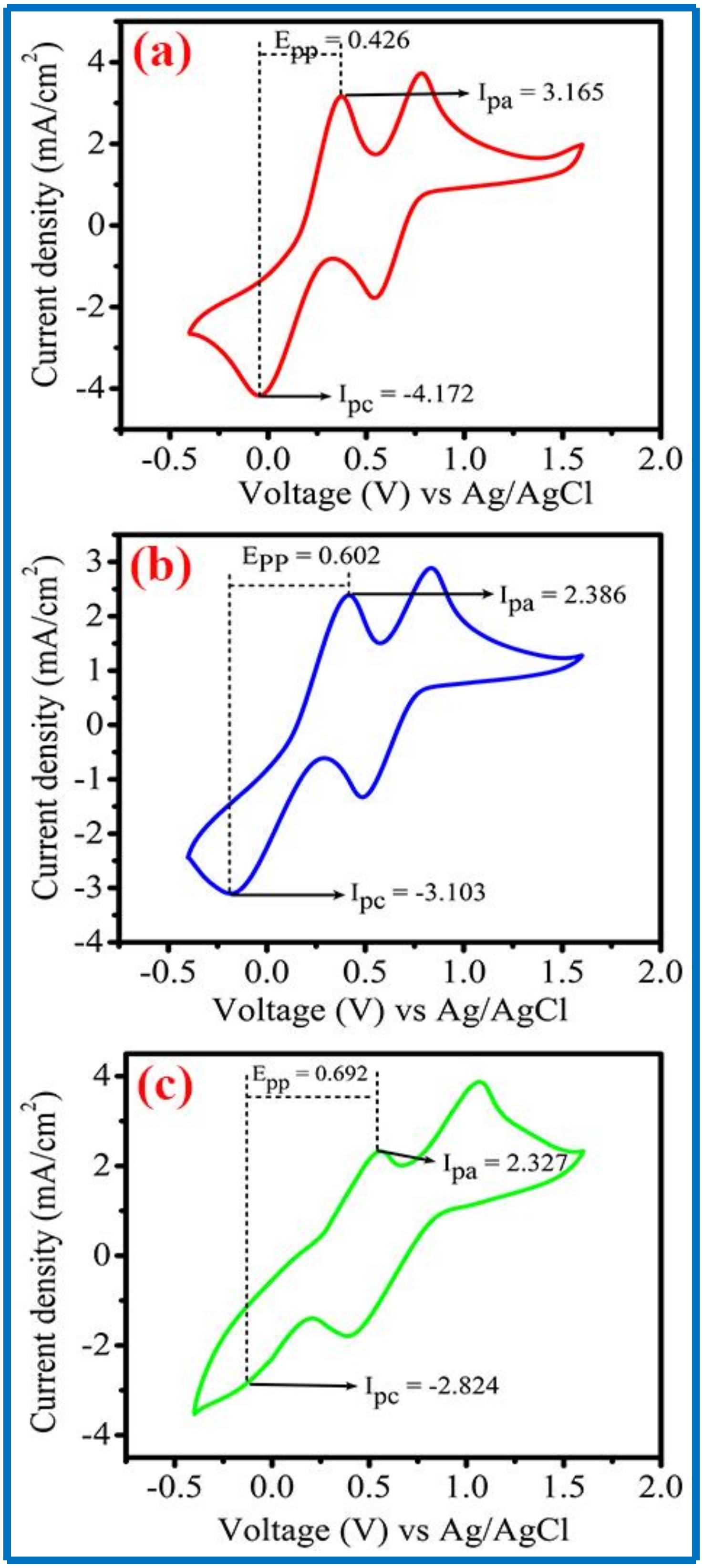

Figure S3. Cyclic voltammetry curves for (a) nano-Si@ACs (b) Pt and (c) ACs.
